# Supplementary material for: The Plasma DIA-Based Quantitative Proteomics Reveals the Pathogenic Pathways and New Biomarkers in Cervical Cancer and High Grade Squamous Intraepithelial Lesion
Source: J Clin Med. 2022 Dec 1;11(23):7155. doi: 10.3390/jcm11237155 (PMC9736146; doi:10.3390/jcm11237155)
Supplement: Supplementary file 1 [file jcm-11-07155-s001.zip › jcm-2011552-Supplementary Figures.pdf]

Supplementary Figures

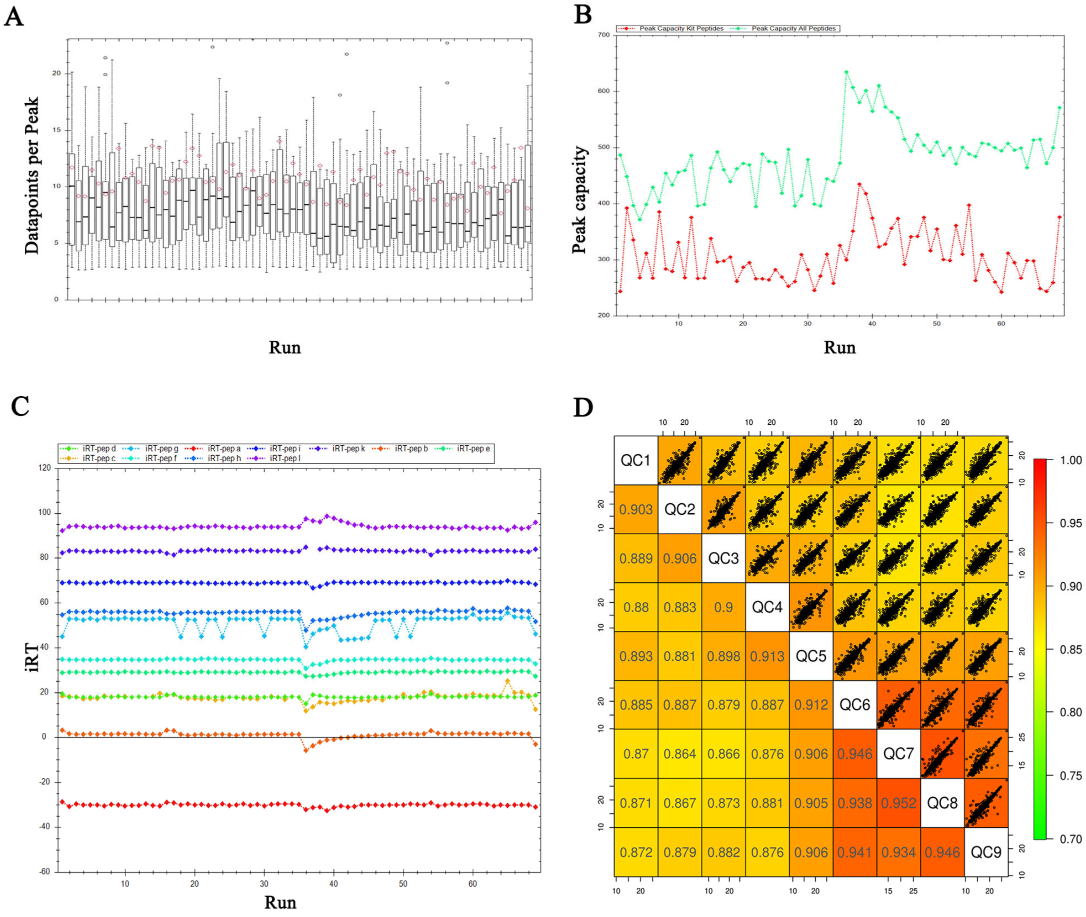

Supplementary Figure S1 Data quality control analysis. (A and B) Datapoints per peak and peak capacity in each DIA run. (C) The iRT points were detected and the retention time of each sample; (D) Intensity correlation of QC samples

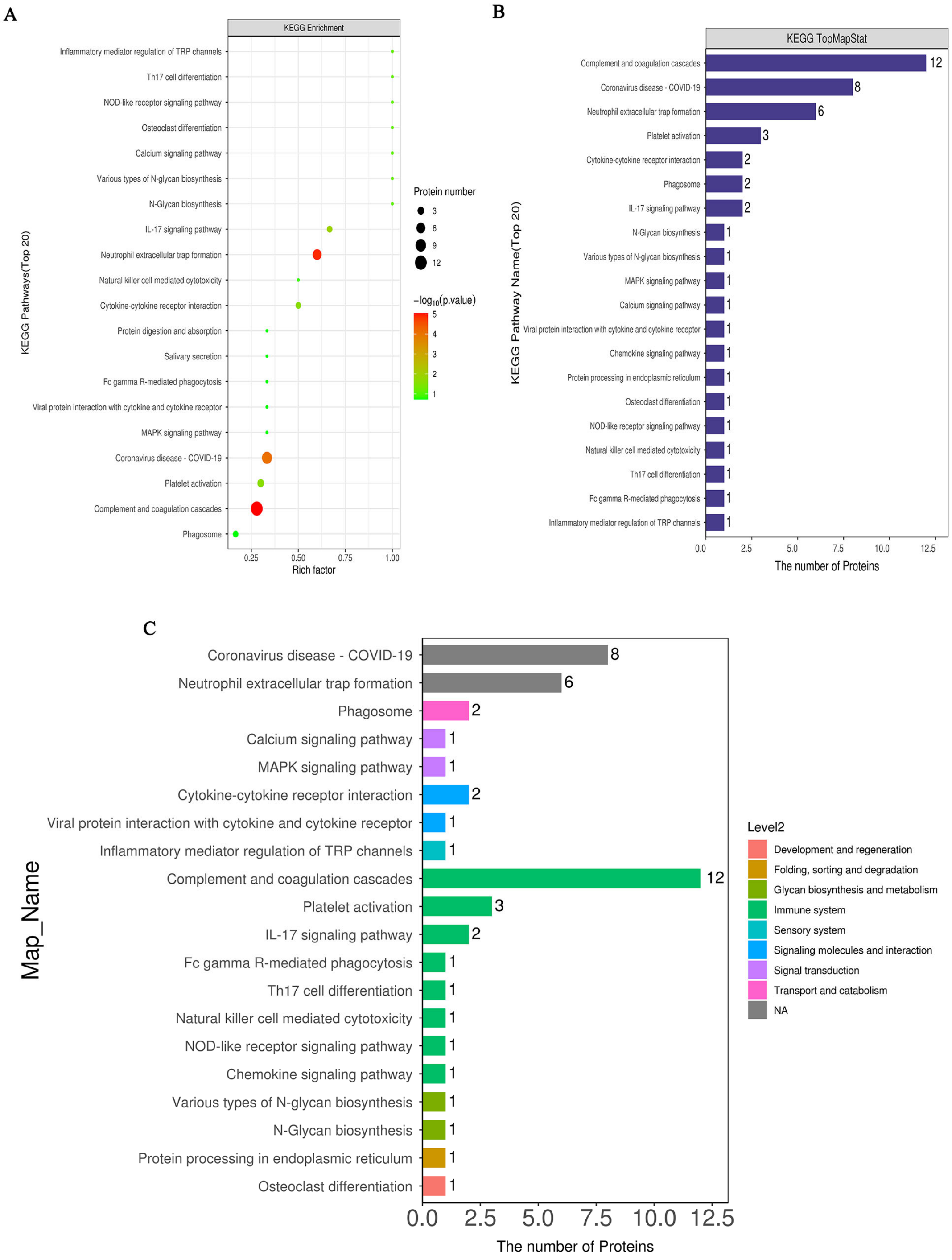

Supplementary Figure S2 The KEGG function enrichment analysis of the green module. (A) The top 20 KEGG pathways; (B) The KEGG pathways with the greatest change of protein numbers; (C) Changes of KEGG secondary pathway.

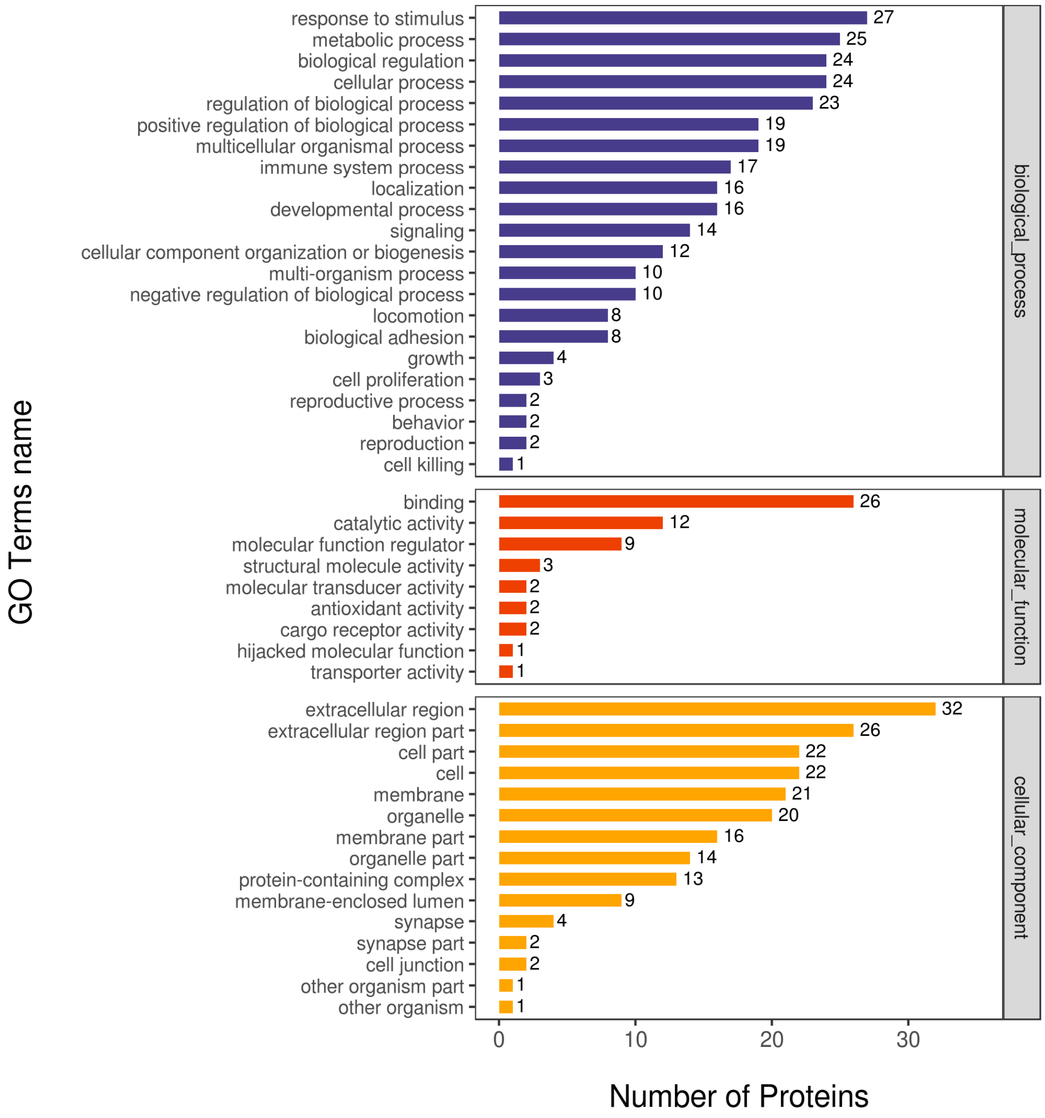

Supplementary Figure S3 GO analysis of the green module.

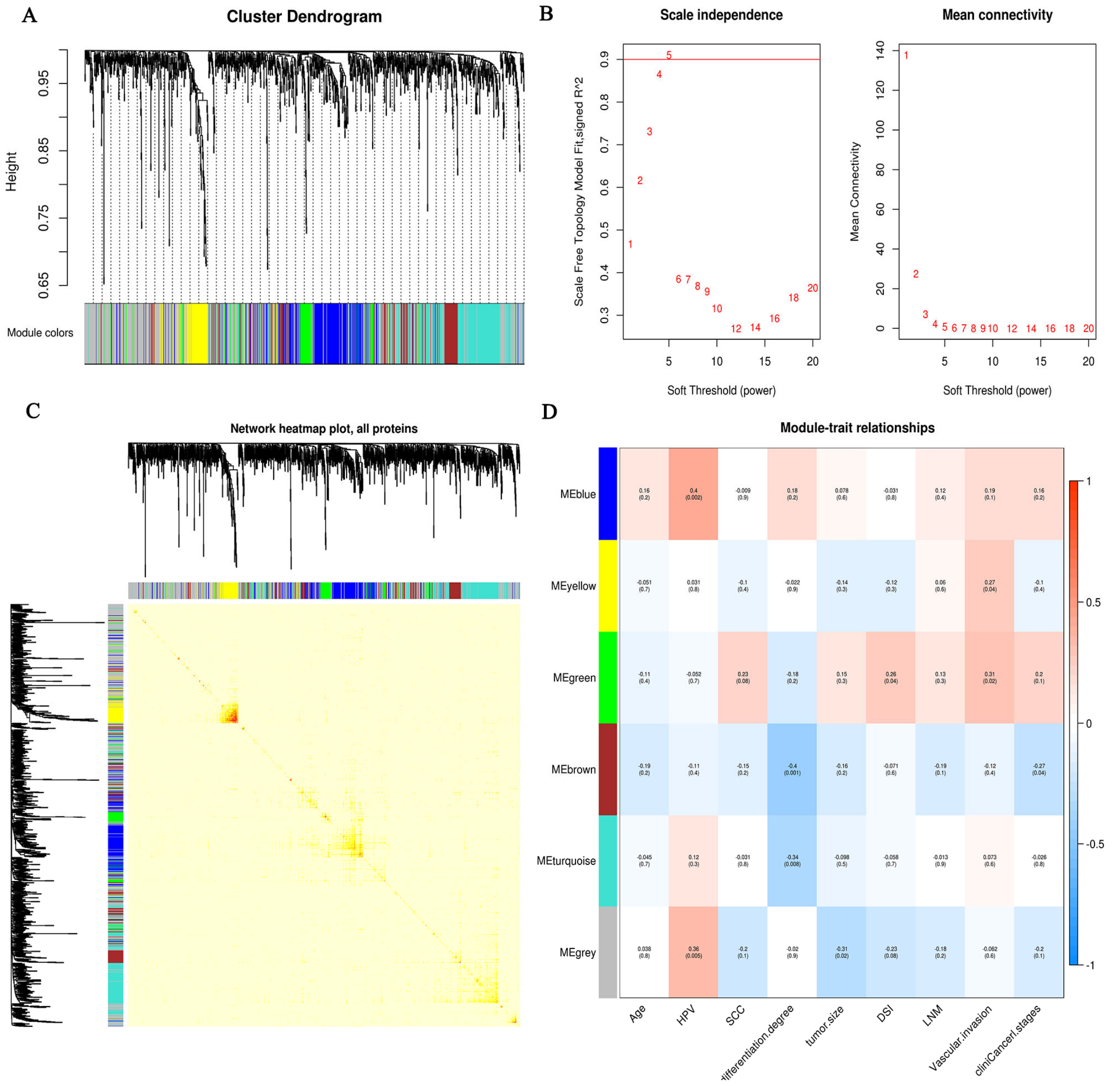

Supplementary Figure S4 WGCNA analysis of the nine clinical clinicopathological factors. (A) Hierarchical clustering tree diagram. (B) Scale-free fit metrics of the network topology obtained using soft threshold analysis; (C) The heat map depicts the difference between all DEPs in the analysis. Light colors indicate low overlap and dark colors indicate high overlap; (D) A total of six co-expression modules were constructed, and each color represents one module of the protein co-expression network constructed by WGCNA. The top numbers in each cell represent Pearson r, and the bottom numbers represent P-values.

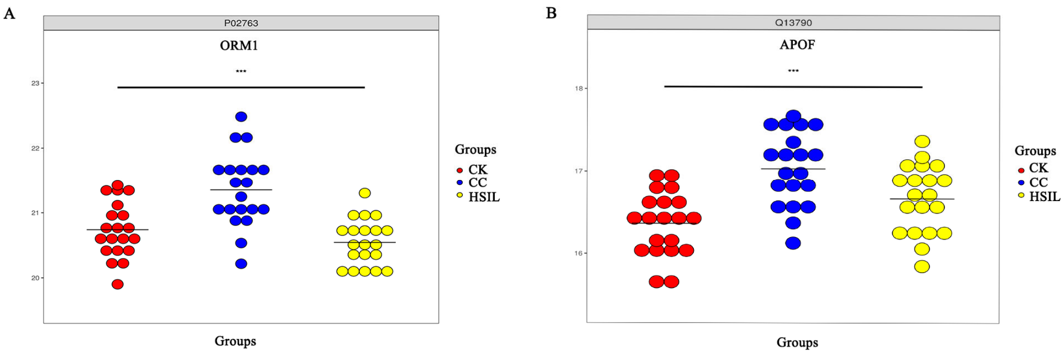

Supplementary Figure S5 The DIA expression pattern of ORM1 and APOF.

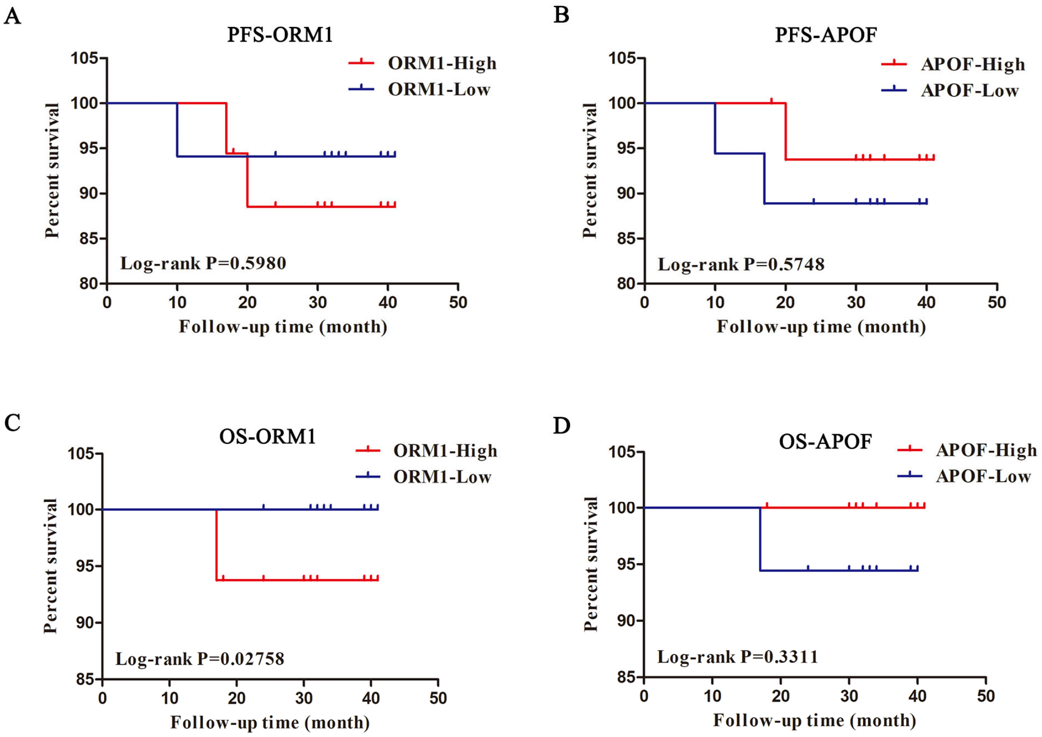

Supplementary Figure S6 (A and B) The PFS of ORM1/APOF high expression cervical cancer patients (50%) compared with the ORM1/APOF low expression (50%); (C and D) The OS of ORM1/APOF high expression cervical cancer patients (50%) compared with the ORM1/APOF low expression (50%).

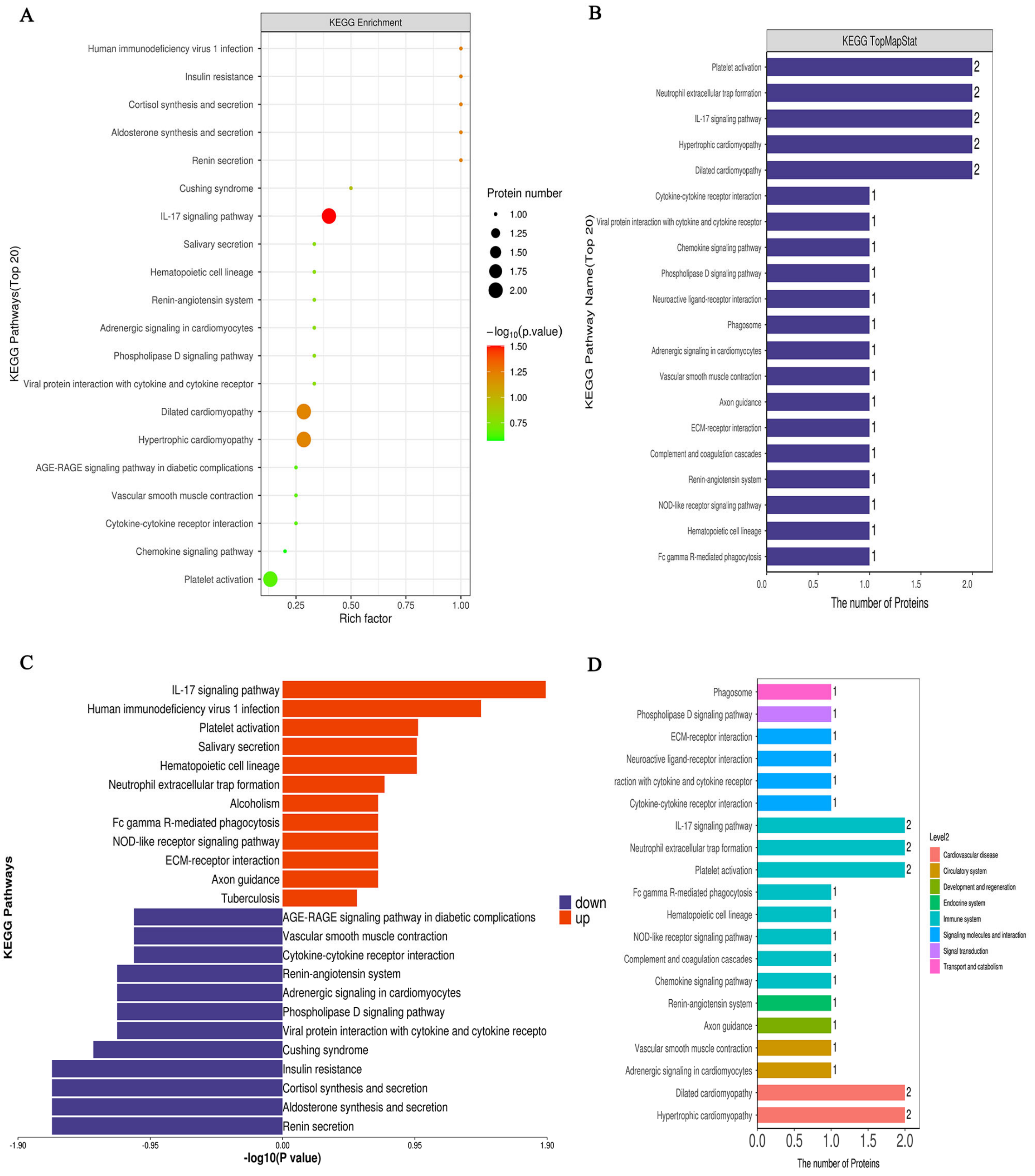

Supplementary Figure S7 The KEGG function enrichment analysis of the CC VS CK. (A) The top 20 KEGG pathways; (B) The KEGG pathways with the greatest change of protein numbers; (C) The changed KEGG pathway was divided into increased group and decreased group; (D) Changes of KEGG secondary pathway.

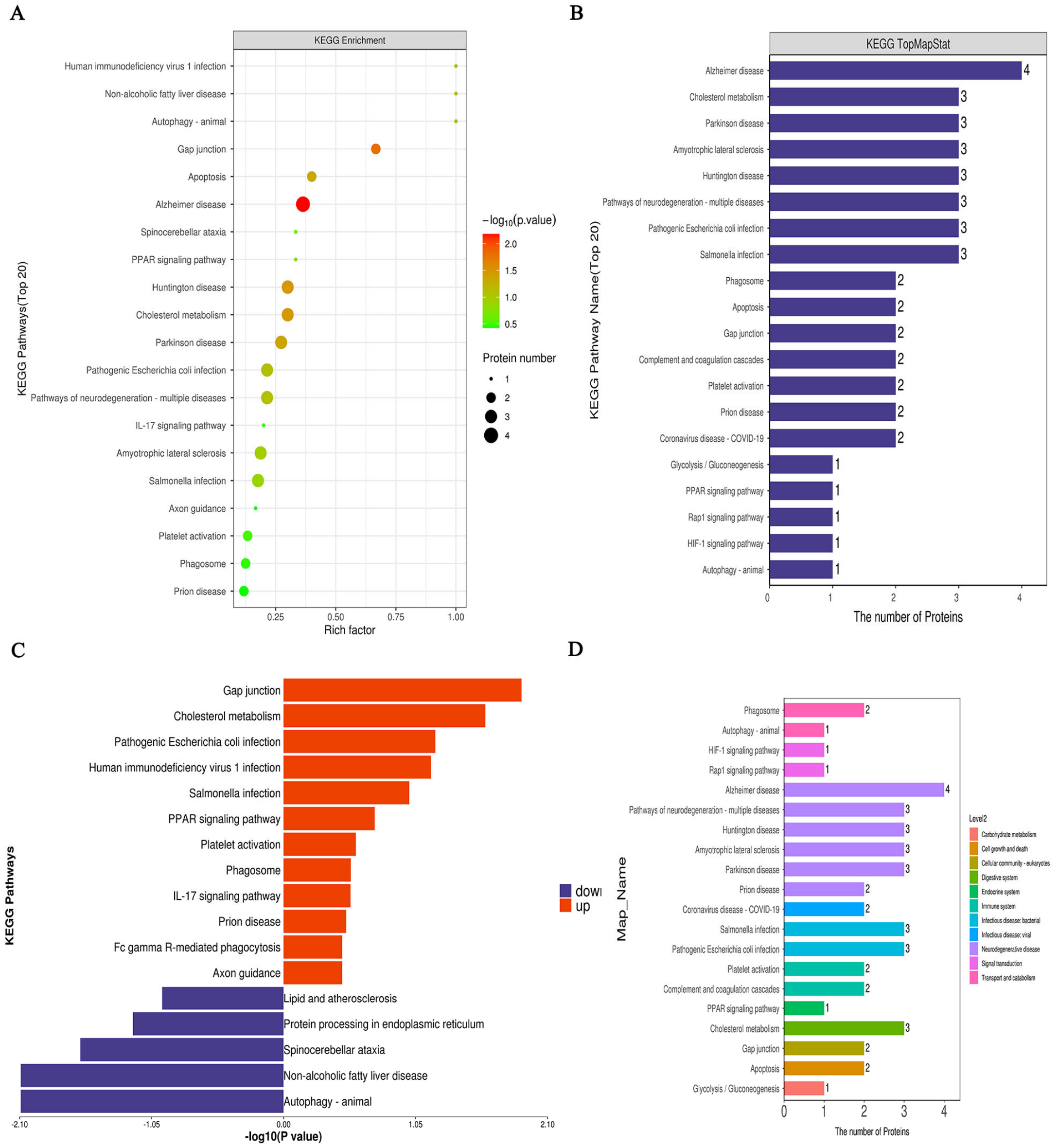

Supplementary Figure S8 The KEGG function enrichment analysis of the HSIL VS CK. (A) The top 20 KEGG pathways; (B) The KEGG pathways with the greatest change of protein numbers; (C) The changed KEGG pathway was divided into increased group and decreased group; (D) Changes of KEGG secondary pathway.

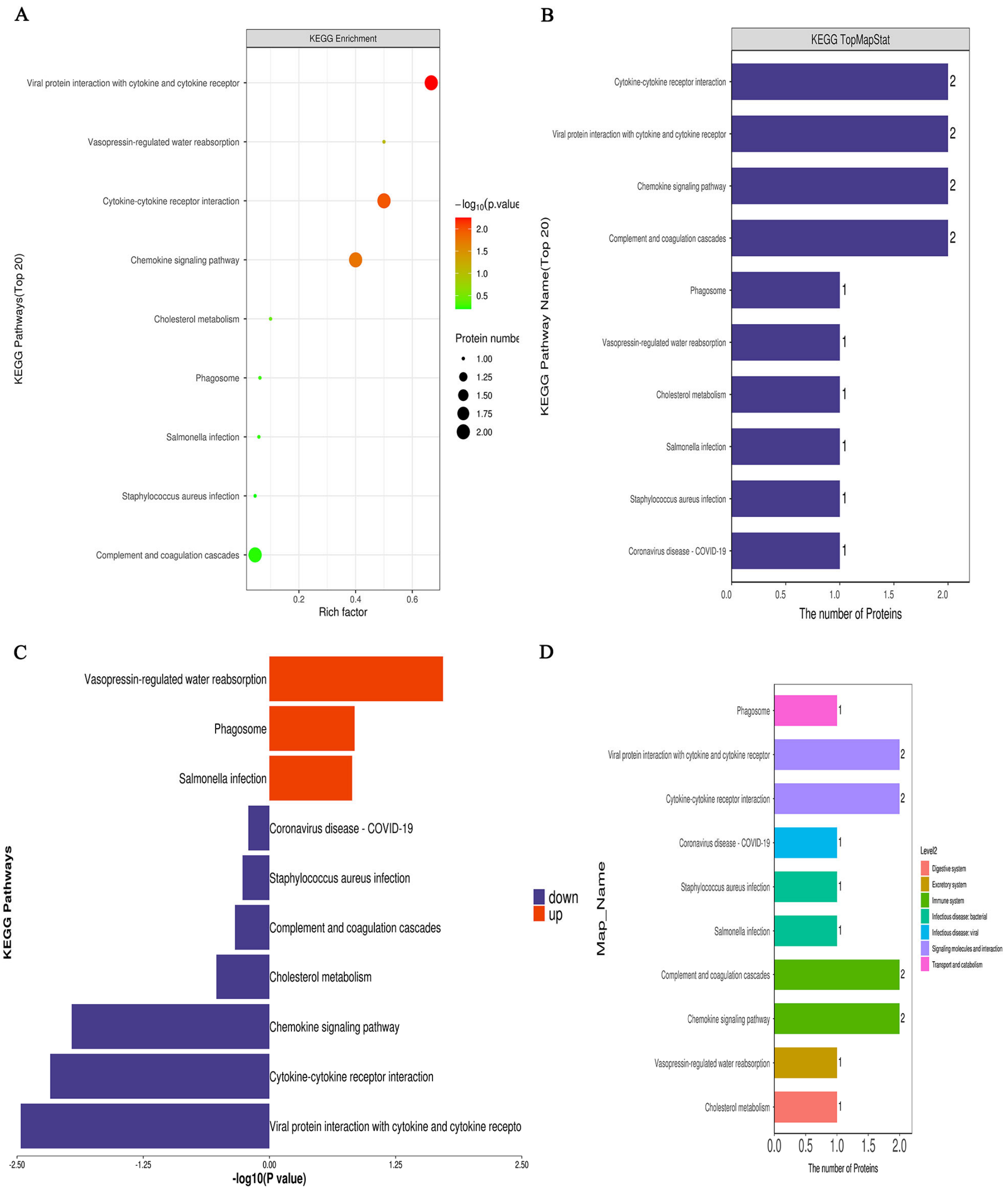

Supplementary Figure S9 The KEGG function enrichment analysis of the CC VS HSIL. (A) The top 20 KEGG pathways; (B) The KEGG pathways with the greatest change of protein numbers; (C) The changed KEGG pathway was divided into increased group and decreased group; (D) Changes of KEGG secondary pathway.

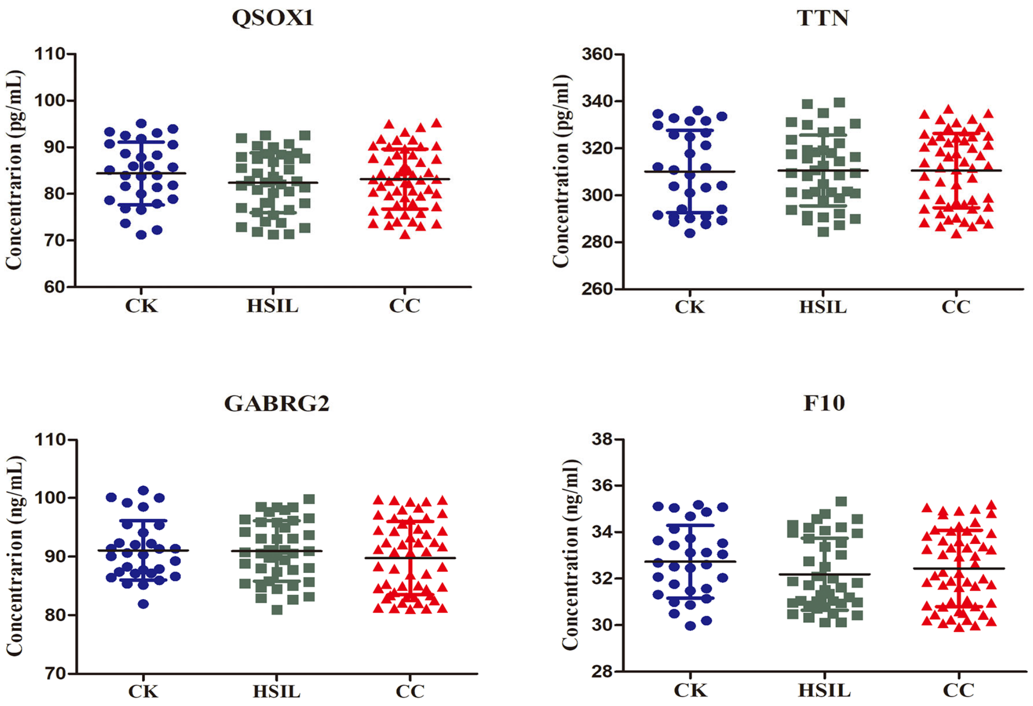

Supplementary Figure S10. The ELISA expression pattern of QSOX1, TTN, GABRG2.
